# TGF-β mimic proteins form an extended gene family in the murine parasite *Heligmosomoides polygyrus*

**DOI:** 10.1016/j.ijpara.2017.12.004

**Published:** 2018-04

**Authors:** Danielle J. Smyth, Yvonne Harcus, Madeleine P.J. White, William F. Gregory, Janina Nahler, Ian Stephens, Edward Toke-Bjolgerud, James P. Hewitson, Alasdair Ivens, Henry J. McSorley, Rick M. Maizels

**Affiliations:** a Wellcome Centre for Molecular Parasitology, Institute of Infection, Immunity and Inflammation, University of Glasgow, UK; b Institute of Infection and Immunology Research and Centre for Immunity, Infection and Evolution, School of Biological Sciences, University of Edinburgh, UK; c MRC Weatherall Institute of Molecular Medicine, University of Oxford, UK; d Centre for Infection and Immunology, Department of Biology, University of York, UK; e MRC Centre for Inflammation Research, Queen’s Medical Research Institute, University of Edinburgh, UK

**Keywords:** TGF-β mimic, *Hp*-TGM, *Heligmosomoides polygyrus*, Helminth, Complement Control Protein family, Regulatory T cell

## Abstract

We recently reported the discovery of a new parasite-derived protein that functionally mimics the immunosuppressive cytokine transforming growth factor (TGF)-β. The *Heligmosomoides polygyrus* TGF-β Mimic (*Hp*-TGM) shares no homology to any TGF-β family member, however it binds the mammalian TGF-β receptor and induces expression of Foxp3, the canonical transcription factor of both mouse and human regulatory T cells. *Hp*-TGM consists of five atypical Complement Control Protein (CCP, Pfam 00084) domains, each lacking certain conserved residues and 12–15 amino acids longer than the 60–70 amino acids consensus domain, but with a recognizable 3-cysteine, tryptophan, cysteine motif. We now report on the identification of a family of nine related *Hp*-TGM homologues represented in the secreted proteome and transcriptome of *H. polygyrus*. Recombinant proteins from five of the nine new TGM members were tested for TGF-β activity, but only two were functionally active in an MFB-F11 reporter assay, and by the induction of T cell Foxp3 expression. Sequence comparisons reveal that proteins with functional activity are similar or identical to *Hp*-TGM across the first three CCP domains, but more variable in domains 4 and 5. Inactive proteins diverged in all domains, or lacked some domains entirely. Testing truncated versions of *Hp*-TGM confirmed that domains 1–3 are essential for full activity in vitro, while domains 4 and 5 are not required. Further studies will elucidate whether these latter domains fulfill other functions in promoting host immune regulation during infection and if the more divergent family members play other roles in immunomodulation.

## Highlights

• A new family of parasite-specific TGF-β mimics was identified.
• Members of the new family are secreted by larval and adult life cycle stages.
• The new family is distantly related to the Complement Control Protein family.
• We experimentally determined active family members and domains within the protein.

## Graphical abstract

**Figure:**
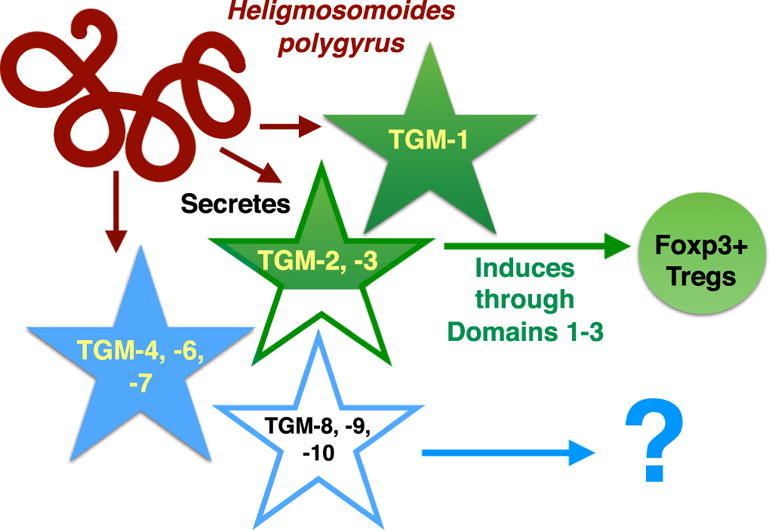

## 1 Introduction

Helminth parasites are well-recognised for their wide-ranging immunomodulatory properties, which in many instances are linked to the stimulation of regulatory T cells (Tregs) that dampen immunity and inflammation (McSorley and Maizels, 2012, Finlay et al., 2014). The murine intestinal helminth *Heligmosomoides polygyrus* is particularly linked to suppression via Tregs (Finney et al., 2007, Setiawan et al., 2007, Rausch et al., 2008, Smith et al., 2016), and mechanistically we showed that parasite secretion of a transforming growth factor (TGF)-β-like protein can account for its ability to expand Treg populations in vivo (Grainger et al., 2010). Notably, TGF-β signalling is also known to be critical to the survival of *H. polygyrus* in its murine host (Ince et al., 2009, Grainger et al., 2010, Reynolds and Maizels, 2012).

TGF-β itself is a member of an ancient gene family shared across all metazoan animals, involved primarily in organismal development. Thus, TGF-β family members are centrally involved in morphogenesis and life cycle progression across all helminth species from free-living nematodes such as *Caenorhabditis elegans* (Ren et al., 1996), to flatworms such as *Schistosoma mansoni* (Freitas et al., 2007, Loverde et al., 2007). In addition, TGF-β homologues produced by some helminth parasites have been reported to act on mammalian receptors (Gomez-Escobar et al., 2000, Sulaiman et al., 2016), and vice versa (Beall and Pearce, 2001, Zavala-Gongora et al., 2006).

Despite this evidence for inter-phylum cross-talk between homologous ligands and receptors, and the identification of the TGF-β homologues encoded by *H. polygyrus* (McSorley et al., 2010), we recently reported that the ability of this parasite to activate the TGF-β pathway resides not with a true homologue, but a structurally novel mimic which replicates the functional activity of this immunosuppressive cytokine (Johnston et al., 2017). The parasite-encoded protein (TGM, for TGF-β Mimic) not only shows no sequence similarity to the host cytokine, it is also considerably larger as an active 404 amino acid (aa) full-length product; in contrast, mature mammalian TGF-β is the C-terminal 110 aa fragment arising from proteolytic processing and activation from a functionally inactive 300 aa precursor.

In our earlier study, it was noted that the full coding sequence of TGM is encoded by 11 exons in the parasite genome; exon I corresponds to a conventional signal sequence, and each successive pair of exons (II + III, IV + V, etc) represents a module or domain of 76–86 amino acids. The five domains of TGM so defined comprise a ∼60 aa sequence with distant similarity to the Complement Control Protein (CCP) domain (Pfam00084), each preceded by a novel tract of 12–15 aa. However, none of the five domains contain all of the CCP consensus residues and they are sufficiently divergent not to return significant homology to other CCP proteins in conventional database searches. The CCP family appears to have undergone extensive radiation and adaptation in many parasitic nematode species (Hewitson et al., 2013), We therefore explored a wider range of TGM-like proteins from *H. polygyrus*, and report below the characterisation of a larger family with nine new products forming a new gene family.

## 2 Materials and methods

### 2.1 Identification of additional secreted TGM family members

A previously published analysis of proteins secreted by adult and L4 stages of *H. polygyrus* had noted a small family of “novel secreted proteins” present in each stage (Hewitson et al., 2013). Subsequently, a separate study matched the TGF-β-like activity from this parasite to one of these novel proteins, named it *Hp-*TGM (GenBank Accession number MG099712) and classified it as an atypical member of the CCP protein family (Pfam00084). Other members of this gene family in *H. polygyrus* were then renamed TGM-2 to TGM-10 as summarised in Table 1, with deduced protein sequences presented in Supplementary Table S1.

**Table 1:** Summary of the extended Transforming Growth factor-β Mimic (TGM) gene family from *Heligmosomoides polygyrus*. Protein sequences are presented in Supplementary Table S1. Genomic analyses were performed on WormBase ParaSite at the Wellcome Trust Sanger Institute, and scaffold numbers relate to the assembly PRJEB1203. Predicted genes with the prefix HPOL correspond to gene models accessible through the same source, and are based on a separate assembly (PRJEB15396).

Gene Name	Length of Protein	NCBI Accession Number	Genomic Scaffold	Predicted Gene	Notes
TGM-1	422	MG099712	0003818	HPOL_0002154401	Predicted protein parts of Exons I, II and VI only, total 132 aa
TGM-2	430	MG429737	0003818	As above	Same locus as TGM-1
TGM-3	429	MG429738	0003818	As above	Same locus as TGM-1
TGM-4	422	MG429739	0000755	None	All 11 exons encoded 77896-86129 of scaffold
TGM-5	341	MG429740	0003818	None	∼3 kb downstream of TGM-1
TGM-6	254	MG429741	0005546	HPOL_0001864701	Predicted 255 aa, 2 aa differences
TGM-7	599	MG429742	0002616	None	
TGM-8	599	MG429743	0006700	None	
TGM-9	252	MG429744	Not found	None	
TGM-10	251	MG429745	0000462	None	

aa, amino acid.

To identify genomic loci, TGM family member sequences were translated and compared (using BLASTP) against both available *H. polygyrus* genomic assemblies produced by the Wellcome Trust Sanger Institute, UK (BioProject PRJEB1203, (International Helminth Genomes Consortium, 2018)) and the Blaxter Laboratory at the University of Edinburgh, UK (BioProject PRJEB15396), both accessible from WormBase ParaSite, https://parasite.wormbase.org (Howe et al., 2016, Howe et al., 2017).

Phylogenetic analysis was performed using MEGA7 (Kumar et al., 2016) and an inferred evolutionary tree drawn using the Neighbour-Joining method (Saitou and Nei, 1987).

### 2.2 Cloning, expression and purification of TGM family members

For protein expression of selected TGM family members, the signal peptide sequence was determined (using the online server SignalP 4.1) and the resulting mature sequence of each (i.e. without signal peptide) was synthesized by GeneArt (Thermo Fisher Scientific, UK) codon-optimised with flanking *Asc*I/*Not*I restriction digest sites and inserted into a holding vector; codon-optimised nucleotide sequences are presented in Supplementary Table S2. Each family member DNA was subsequently subcloned into mammalian expression vector pSECTag2A (Invitrogen, USA) using restriction sites *Asc*I and *Not*I, and resultant constructs were sequence verified.

Ten micrograms of purified plasmid DNA of each construct was transiently transfected into HEK293T cells using the calcium chloride method (Promega, USA). Cells were switched into serum-free media (293 SFM II; Gibco, USA) after 24 h, and cultured for up to 4 days, after which point supernatants were collected, filtered through a 0.45 µM filter and dialysed into imidazole-free binding buffer before being loaded onto a 1 ml nickel sulphate charged HiTrap Chelating HP column (GE Healthcare, USA) using a peristaltic pump. Proteins were purified using an Akta Purifier (GE Healthcare) and positive fractions were dialysed into PBS.

### 2.3 Cloning of truncated Hp-TGM constructs

Mammalian codon-optimised *Hp*-TGM was synthesized by GeneArt as previously published (Johnston et al., 2017), inserted into a holding vector and subcloned into the mammalian expression vector pSECTag2a using restriction sites *Asc*I and *Apa*I.

Amplification and cloning of the truncated versions of *Hp*-TGM was performed by PCR amplification using full-length codon-optimised *Hp*-TGM (Supplementary Table S2) as template DNA and domain-specific primers with restriction sites (*Asc*I/*Apa*I) and cap sequence (gcgcgc) placed at either end (see Supplementary Table S3). Primer pairs were used to systematically truncate one or more domains either from the N-terminus or C-terminus with the resultant nomenclature being TGMΔ1 = TGM with domain 1 truncated; TGM Δ1,2 = TGM with domains 1 and 2 truncated, etc.

Truncated *Hp*-TGM inserts were amplified using proofreading Taq polymerase Phusion Hi Fidelity Taq polymerase (New England Biolabs (NEB), USA) under the following conditions: Initial denaturation at 98 °C for 30 s followed by 35 cycles of denaturing at 98 °C for 30 s; annealing at 55–65 °C for 30 s; extension at 72 °C for 30–90 s (depending on truncation size) with a final extension of 72 °C for 10 min and 12 °C hold. PCRs were electrophoresed through a 1.2% agarose gel and a single band of each predicted size of insert was gel extracted, cloned into a pSECTag2A vector (Invitrogen) and transformed into JM109 bacterial cells, selecting with Ampicillin (100 µg/ml). Transformants were sequence checked before transfection and expression in HEK293T cells.

### 2.4 Functional assays

#### 2.4.1 TGF-β bioassay

The TGF-β bioassay (cell line clone MFB-F11) developed by Tesseur et al. (2006) was performed as previously described (Johnston et al., 2017). MFB-F11 cells were tested and found to be mycoplasma-free. Briefly, confluent cells were detached with trypsin, and resuspended in DMEM with 2.5% FCS, 100 U/ml of penicillin, 100 μg/ml of streptomycin and 2 mM L-glutamine at a concentration of 4x10^5^ cells/ml. In 50 μl, 4x10^4^ cells were added to each well of a 96-well round-bottomed plate. HEK293T cell supernatants (for truncated *Hp*-TGM proteins) or log dilutions (starting at 1000 ng/ml) of purified proteins such as *Hp-*TGM or TGM family members were then added to each well in a volume of up to 50 μl and incubated for 24 h at 37 °C. For TGF-β receptor 1 and 2 inhibitor assays, either 5 µM SB431542 (Tocris Bioscience, UK) or 10 µM ITD-1 (Tocris Bioscience) were added to each well with DMSO added to control wells. For anti-TGM assays, rat polyclonal anti-*Hp*-TGM antibody was produced as previously described (Johnston et al., 2017) and added at a final concentration of 10 µg/ml with purified rat IgG used as a control at the same concentration (purified from the serum of Wistar rats in–house). Subsequently, 20 μl of supernatant were aspirated from each well, added to an ELISA plate (Nalge Nunc International, USA) with 180 μl of reconstituted Sigma FastTM p-nitrophenyl phosphate substrate and incubated at room temperature in the dark for up to 4 h. Plates were read on at 405 nm on an Emax precision microplate reader (Molecular Devices, USA). All conditions were set up in duplicate and repeated at least twice.

#### 2.4.2 Foxp3^+^ Treg induction assay

A single cell suspension was prepared from the spleens of naïve Foxp3-GFP BALB/c transgenic mice (Fontenot et al., 2005), with contaminating red blood cells removed by resuspending the cells from one spleen in 2 ml of red blood cell lysis buffer (Sigma) and incubating at room temperature for 2 min. Cells were then washed and resuspended in DMEM containing HEPES (Gibco), supplemented with 2 mM L-glutamine, 100 U/ml of penicillin and 100 μg/ml of streptomycin (Gibco), 10% heat-inactivated FCS (Gibco), and 50 nM 2-mercaptoethanol (Gibco). CD4^+^ T cells were enriched for by magnetic sorting using the mouse CD4^+^ T cell isolation kit on the AutoMACS system (Miltenyi, Germany) as per the manufacturer’s instructions. Cells were cultured at 1x10^5^ per well in flat-bottomed 96-well plates (Corning, USA) with the addition of IL-2 (Miltenyi) at a final concentration of 200 U/ml and anti-CD3/anti-CD28 coated beads (Miltenyi) at a bead to cell ratio of 1:4. Purified proteins (eg. TGF-β, *Hp*-TGM, TGM family members) were added in log dilutions starting at 100 ng/ml and cultured at 37 °C in 5% CO_2_ for at least 72 h before being removed for flow cytometric analysis. All conditions were set up in triplicate and repeated at least twice.

#### 2.4.3 Flow cytometric analysis

For viability staining, LIVE/DEAD® fixable blue (Life Technologies, USA) was diluted to 1:1000 in PBS; 100 μl were added to each sample of cells, which was then incubated in the dark for 20 min at 4 °C and washed twice in FACS buffer (1 x PBS, 0.5% (w/v) BSA, 0.05% sodium azide). To prevent non-specific antigen binding, cells were incubated with 50 µl of polyclonal IgG (Sigma) (diluted 1:50 in FACS buffer) for 10 min at 4 °C and then washed twice in FACS buffer. Cells were stained with anti-CD4–PerCP/Cy5.5 (GK1.5, Biolegend, USA) to a total volume of 50 µl (diluted 1:200). Separate Foxp3 staining was not required as cells were from Foxp3-GFP transgenic mice. Single stain controls were individually added to one drop of UltraComp eBeads (eBioscience, USA). Samples were incubated for 20 min at 4 °C, washed twice in FACS buffer and then resuspended in 300 ul of FACS buffer. All samples were acquired on a BD Biosciences LSR Fortessa flow cytometer or Celesta and analysed using FlowJo software (Tree Star). The gating strategy is provided in Supplementary Fig. S1.

#### 2.4.4 Ethical approval and care of animals

Mouse cells isolated for Foxp3*^+^* Treg induction assays (Section 2.4.2) were from spleens of inbred BALB/c Foxp3-GFP reporter mice (Fontenot et al., 2005) aged 6–12 weeks, bred in–house and maintained in individually ventilated cages under conventional conditions. All animal breeding was performed under UK Home Office licence and approved by the University of Glasgow Ethical Review Board.

## 3 Results and discussion

### 3.1 Identification of a wider TGM gene family in *H. polygyrus*

**Fig. 1:**
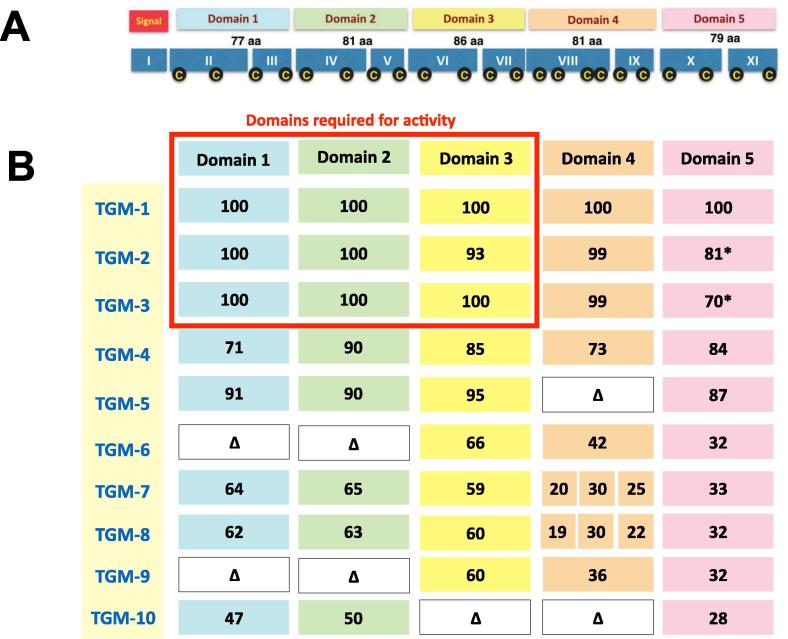
Extended family of secreted transforming growth factor (TGF)-β mimic (TGM) proteins from *Heligmosomoides polygyrus.* TGM-like proteins secreted by adult and larval stages of *H. polygyrus*, and their similarity (% amino acid identity) to each of the five domains of TGM. Missing domains are shown as empty boxes and the Δ symbol. * in Domain 5 denotes an 8 amino acid (aa) C-terminal extension is present (EGYSLILE in TGM-2, EEYFLILE in TGM-4). Note that TGM-7 and -8 include two additional domains inserted after domain 3. The red outline denotes the domains and proteins found to show functional mimicry of transforming growth factor (TGF)-β.

TGM was discovered by matching mass spectrometric peptide fragmentation data to an in–house transcriptome database assembled from high-throughput mRNA sequencing (Hewitson et al., 2013, Johnston et al., 2017). In following up these studies, nine additional proteins secreted by *H. polygyrus* were identified which had significant sequence similarity with the original TGM (termed here TGM-1) as summarised in Fig. 1. From adult parasites, three closely related variants were found and named TGM-2 (95.8% amino acid identity across the mature protein), TGM-3 (93.8%) and TGM-4 (80.4%). In addition, two more variants were found in the adult secretome which lacked entirely, and exactly, domain 4 (TGM-5, with 90.1% identity across the other four domains) or domains 1 and 2 (TGM-6, with 47.2% identity across the remaining three domains). Finally, four homologues (TGM-7 to -10) were secreted by the larval stage, as detailed below. These discoveries indicated that the putative domains were discrete subunits which could be added or subtracted from the protein without necessarily compromising structure or solubility.

Three other adult-associated TGM homologues mapped to separate loci in the genome, which correspond to the complete sequences of TGM-4 and TGM-6; in addition, exons I-V (ie the signal peptide and domains 1 + 2) of TGM-5 mapped ∼ 3.3 kb downstream of TGM at the end of the existing scaffold in the assembly (Table 1). Only TGM-6 is represented by an accurate gene model in the current rendition of the predicted proteome (Table 1).

In our earlier study (Hewitson et al., 2013), four proteins secreted by the tissue-dwelling L4 stage of the parasite had been found with sequence homology only to the transcript subsequently identified as TGM-1; each of these were highly abundant in the larval secretome, and had been named Larval Secreted Protein (LSP) -1, -2, -3 and -6 according to their overall abundance ranking among all novel secreted proteins. These were renamed TGM-7 to TGM-10, respectively. Notably, TGM-7 and TGM-8 contained an additional two CCP domains, rendering both proteins 599 aa in length, while TGM-9 and -10 each have lost two domains (corresponding to domains 1 + 2, and 3 + 4, respectively). These findings again emphasise the plasticity of domain gain and loss in the new gene family. Details of the amino acid sequences for all 10 family members are given in Supplementary Table S1.

**Fig. 2:**
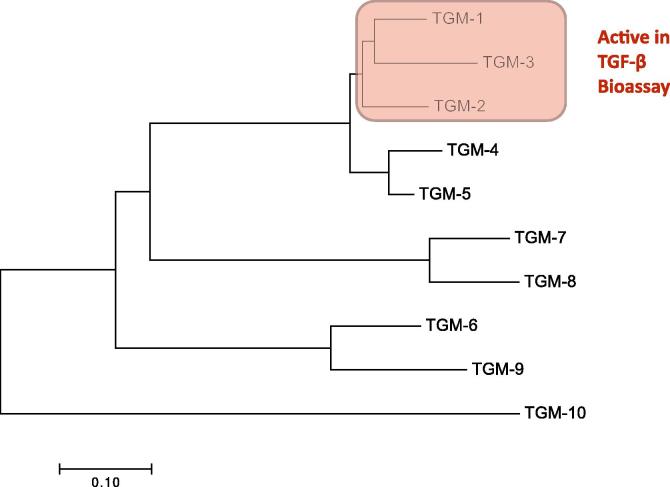
Phylogenetic tree of the transforming growth factor (TGF) -β mimic (TGM) family members in *Heligmosomoides polygyrus*. The 10 amino acid sequences of TGM family members presented in Table 1 were aligned by MEGA7 using the Neighbour-Joining method as cited in the Section 2.1. The tree is drawn to scale with branch lengths indicating inferred evolutionary distances.

Based on the domain model for TGM-1 presented in Fig. 1, it is evident that domains 1–3 are more closely conserved, while domain 5 is the most variable, and that the gene family displays plasticity in the number of CCP domains per protein. Although the diversity in both sequence and length across the broader family of TGM-like proteins was quite extensive, a phylogenetic tree could be constructed (Fig. 2).

Mapping of the deduced protein sequences with the genome assembly of *Heligmosomoides polygyrus bakeri* available through the Sanger Institute at WormBase ParaSite (https://parasite.wormbase.org, Howe et al., 2016, Howe et al., 2017), indicated that TGM-1, -2 and -3 all correspond to the same genomic segment (Table 1) and may thus be allelic forms of the same locus; however, none match the genome sequence completely. As the parasite is in effect an outbred strain, and samples for sequencing were taken at different times, exact matching between transcriptome and genome assemblies will not always occur. Annotation of the open reading frame of the TGM-1 locus, with particularly small ∼70-nucleotide (nt) exons III, V, VII, IX and XI may be problematic with gene prediction algorithms, as only a small proportion of the TGM-1 protein sequence was found to be represented by a gene model in the current *H. polygyrus* genome assembly (Table 1).

### 3.2 Functional testing of TGM gene family members

**Fig. 3:**
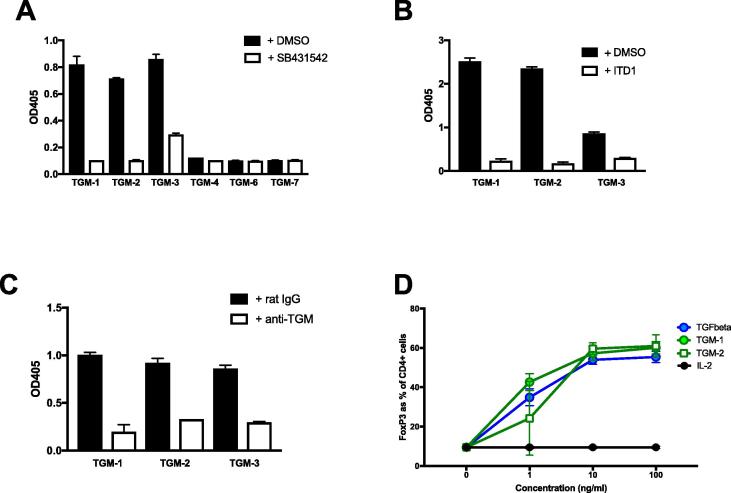
Functional testing of *Heligmosomoides polygyrus* TGM transforming growth factor (TGF) -β mimic (TGM) family members. Proteins were tested functionally in the MFB-F11 bioassay (fibroblast cell line isolated from mouse embryos lacking TGF-β). (A–C) or with murine T cell induction of Foxp3 expression (D). TGM-5 is omitted from the assays as it was not possible to express recombinant protein in the same system as the other family member proteins. (A) Activity detected in the MFB-F11 bioassay after 24 h of culture with 100 ng/ml of TGM family members TGM-1, -2, -4 and -6, and 10 ug/ml of TGM-3 in the absence or presence of the type I transforming growth factor (TGF)-β receptor kinase inhibitor, SB431542. (B) Activity in the same bioassay of the family members found positive in A, in the absence or presence of the type II TGF-β receptor inhibitor, inducer of type II TGF-β receptor (TGFBR2) degradation-1 (ITD-1) (10 µM). (C) Activity in the bioassay of family members in the absence or presence of anti-TGM-1 antibody (10 µg/ml) or rat IgG control (10 µg/ml). (D) Percentage of Foxp3 induction induced by TGM-1 and TGM-2 compared with IL2 only (no induction control) and TGF-β (positive control).

To test the functional capacity of the sequence variants within the TGM family, we first used a sensitive bioassay with MFB-F11 cells, which express alkaline phosphatase under the control of a Smad-driven promoter. Notably TGM-2 and -3, similar to TGM-1, showed strong activity which was blocked by the TGF-β signalling inhibitor SB431542. However, the TGM-4 and TGM-6 proteins were not active in the same assay (Fig. 3A). Attempts to express recombinant TGM-5 protein were unsuccessful, so the functional properties of this variant remain to be determined. For the larval TGM family members, TGM-7 was expressed and tested in the same assays but proved to be inactive (Fig. 3A).

To verify that the active proteins were engaging the TGF-β signalling pathway, the inhibitor ITD-1 which promotes degradation of the TGFβ receptor (Willems et al., 2012) was shown to block activation of MFB-F11 cells by TGM-1, -2 and -3 (Fig. 3B). In addition, rat polyclonal antibody raised against TGM-1 strongly inhibited the ability of all three isoforms to activate the cell line (Fig. 3C). As TGM-2 differs from TGM-1 in domains 3–5 (Fig. 1), its ability to also induce the Treg transcription factor Foxp3 in CD4^+^ T cells was tested. Indeed, TGM-2 showed almost identical activity as TGM-1 in inducing this transcription factor in naive, Foxp3-negative cells in vitro (Fig. 3D).

### 3.3 Assignation of functional domains

**Fig. 4:**
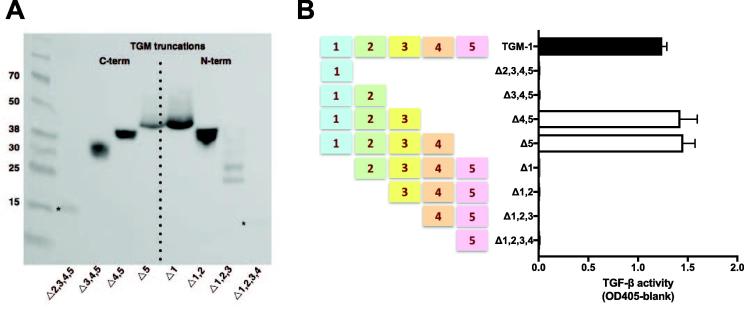
Truncation analysis of *Heligmosomoides polygyrus* transforming growth factor (TGF) -β mimic (TGM) domains. Functional testing of N- and C-terminal (term) truncations of TGM-1 to determine essential domains for biological activity. (A) Anti-6xHis western blot of transiently expressed TGM truncations in HEK293T cells to show protein expressed before adding supernatants to the MFB-F11 bioassay (fibroblast cell line isolated from mouse embryos lacking transforming growth factor (TGF)-β). * indicates a faint band present on the blot. (B) Activity profile of TGM truncations in the MFB-F11 reporter cell assay for TGF-β activity. A volume of 50 µl was added of each truncation supernatant. Full-length TGM-1 supernatant was used as a positive control.

Comparison among the active homologues indicated that conservation of domains 1–3 was shared by the functional proteins (Fig. 1). On the strength of these observations, and to further delineate the functional and non-functional domains of TGM-1, a range of truncation constructs were expressed (as shown in Fig. 4A) to experimentally determine the domains required for full activity. Each truncation was constructed by PCR amplification from the full-length open reading frame DNA of codon-optimised TGM-1, using specific primers. Plasmids containing verified truncated sequence were used to transform HEK293T cells for expression. Transiently expressing HEK293T culture supernatants were used to test for activity in the MFB-F11 reporter cell assay for TGF-β activity. As shown in Fig. 4B, the minimal effective structure of active TGM-1 was found to be domains 1–3, with domains 4 and 5 found not to be necessary for full activity, and the absence of domain 1 abolishing biological activity.

**Fig. 5:**
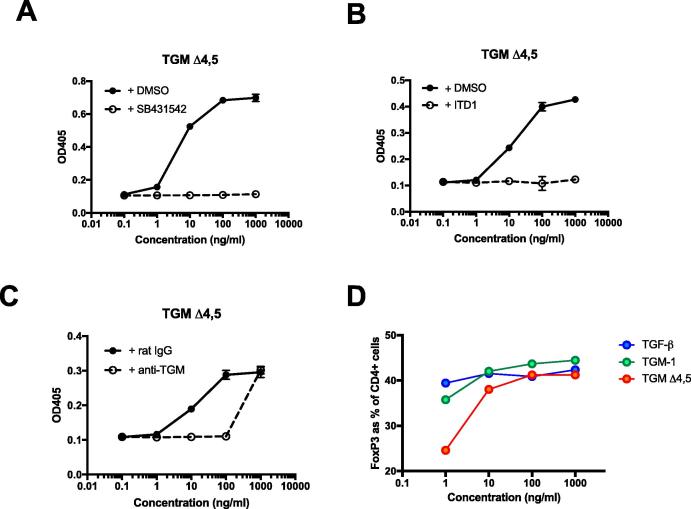
Functional testing of recombinant truncated *Heligmosomoides polygyrus* transforming growth factor (TGF) -β mimic (TGM)Δ4,5. Recombinant truncated TGMΔ4,5, comprising domains 1–3 alone, was tested functionally in the MFB-F11 bioassay (fibroblast cell line isolated from mouse embryos lacking TGF-β). (A–C) and for murine T cell induction of Foxp3 expression (D). (A) Activity detected in the MFB-F11 bioassay after 24 h of culture with log fold dilutions of TGM Δ4,5 starting at 1000 ng/ml, in the absence or presence of the type I transforming growth factor (TGF)-β receptor inhibitor, SB431542. (B) As (A) but in the absence or presence of the type II TGF-β receptor inhibitor, ITD-1 (10 µM).(C). As (A) but in the presence of either polyclonal rat anti-TGM antibody (10 µg/ml) or rat IgG control (10 µg/ml). (D) Percentage of Foxp3 induction induced in naive Foxp3-GFP negative splenic CD4^+^ T cells in response to TGM Δ4,5 compared with TGF-β and TGM (positive induction controls).

The minimal active form of TGM-1 was thus found to be the Δ4,5 form (TGM-Δ4,5) which was further tested, showing full inhibition by the inhibitors ITD-1 (Fig. 5A), and SB432542 (Fig. 5B), consistent with the properties of the full-length protein. In addition, activity was neutralised by polyclonal anti-TGM antibody (Fig. 5C). Finally, TGM-Δ4,5 was tested in cultures of naive murine T cells for Foxp3 induction and found to be very effective at all but the lowest concentrations compared (Fig. 5D).

## 4 Conclusion

Most pathogens have evolved sophisticated molecular strategies to block or redirect host immunity, thereby ensuring their survival in the host (Alcami and Koszinowski, 2000, Baxt et al., 2013). Among parasitic helminths, a broader immune suppression of both parasite and bystander antigens is often observed (Yazdanbakhsh et al., 2002), and can be attributed in part at least to the expansion of regulatory T cells through the TGF-β pathway (McSorley and Maizels, 2012). Thus, our earlier reports that the murine nematode *H. polygyrus* secretes a ligand which activates TGF-β signalling (Grainger et al., 2010, Johnston et al., 2017) provide a clear example of parasite-mediated manipulation of host immunoregulatory pathways to promote its own survival.

Unexpectedly, the identification of the parasite ligand revealed it to be not related to the TGF-β family (although that is well represented in helminth parasites), but a novel protein resulting from adaptation of a very different evolutionary module, the CCP domain. However, the TGMs diverge from the consensus at several conserved residue positions, and include atypical insertions which extend each domain significantly. Interestingly, family members with domain deletions and insertions map to different loci in the *H*. *polygyrus* genome assemblies, indicating that these variants are evolutionarily diverging genes rather than alternatively spliced forms encoded at a single locus.

The CCP, or Sushi, domain is an ancient motif from the origins of the animal kingdom (King et al., 2003), and hence is well represented in the genomes of all parasitic helminths thus far sequenced. In *H. polygyrus* there is a larger family of CCP proteins, which includes a recently reported Alarmin Release Inhibitor (Hp-ARI). Hp-ARI is a 3-domain product that binds the epithelial cell alarmin, IL-33, thereby blocking sensitization and type 2 innate lymphoid cell responses involved in initiation of the type 2 immune response (Osbourn et al., 2017). It is striking that ARI and TGM target such different pathways, key to the innate and adaptive immune responses, respectively, and yet both appear to have evolved from the same ancestral building block. CCP domain proteins are highly expressed in many other parasites including those of humans and it will be important to survey these for similar, or new, immunomodulatory activities.

In this report we now show that *H. polygyrus* expresses and secretes a set of TGM-like proteins with substantial plasticity in domain structure and sequence. Preliminary inspection of the parasite genome sequence indicates that in most cases, domain gain and loss resulted from gene duplication and modification, although at this stage we cannot exclude alternative splicing events which are known to be widespread both in helminths (Shoemaker et al., 1992, Chalmers et al., 2008, Zahler, 2012) and in protozoan parasites (Lunghi et al., 2016). More intriguing is the question of functional diversification within the TGM family, and whether the homologues that do not directly mimic TGF-β have evolved quite different functions within the overall setting of parasite modulation of host immunity.
