# Supplementary material for: TGF-β mimic proteins form an extended gene family in the murine parasite Heligmosomoides polygyrus
Source: Int J Parasitol. 2018 Apr;48(5):379–85. doi: 10.1016/j.ijpara.2017.12.004 (PMC5904571; doi:10.1016/j.ijpara.2017.12.004)
Supplement: Supplementary Table S1 [file mmc1.docx]

**Supplementary Table S1**

Amino acid sequences of *Heligmosomoides polygyrus* Transforming Growth factor-β Mimic (TGM) family members described in Table 1 and elsewhere in this report.

| Gene Name | NCBI Accession  Number | Amino Acid Sequence (stop codon denoted by *) |
| --- | --- | --- |
| **TGM-1** | MG099712 | MLLTVVIGLLEVAATDDSGCMPFSDEAATYKYVAKGPKNIEIPAQIDNSGMYPDYTHVKRFCKGLHGEDTTGWFVGICLASQWYYYEGVQECDDRRCSPLPTNDTVSFEYLKATVNPGIIFNITVHPDASGKYPELTYIKRICKNFPTDSNVQGHIIGMCYNAEWQFSSTPTCPASGCPPLPDDGIVFYEYYGYAGDRHTVGPVVTKDSSGNYPSPTHARRRCRALSQEADPGEFVAICYKSGTTGESHWEYYKNIGKCPDPRCKPLEANESVHYEYFTMTNETDKKKGPPAKVGKSGKYPEHTCVKKVCSKWPYTCSTGGPIFGECIGATWNFTALMECINARGCSSDDLFDKLGFEKVIVRKGEGSDSYKDDFARFYATGSKVIAECGGKTVRLECSNGEWHEPGTKTVHRCTKDGIRTL* |
| **TGM-2** | MG429737 | MLLTVVIGLLEVAATDDSGCMPFSDEAATYKYVAKGPKNIEIPAQIDNSGMYPDYTHVKRFCKGLHGEDTTGWFVGICLASQWYYYEGVQECDDRRCSPLPTNDTVSFEYLKATVNPGIIFNITVHPDASGKYPELTYIKRICKNFPTDSNVQGHIIGMCYNAEWQFSSTPTCPASGCPPLPDDGIVFYEYYGYAGDRHTVGPVVTKDSSGNYPSPTHARRRCRALSQKADTGEFVAICYKSCTTGESHWQYYKYIKNCPDPRCKPLKADESVRYEYFTMANETGKKEGTPAQVDKGGKYSQHTCVRKFCDKSPYTCSVKGPIFGECLDGQWNFTALDECLNARGCDGGDLFNKLGFEIVMVREGEGSDSYKDDYVRFYTTGSKVNAECKGKTVRLECSNGEWHDSETRTVHRCTSEGIRHYEGYSLILE* |
| **TGM-3** | MG429738 | MLLTVVIGLLEVAATDDSGCMPFSDEAATYKYVAKGPKNIEIPAQIDNSGMYPDYTHVKRFCKGLHGEDTTGWFVGICLASQWYYYEGVQECDDRRCSPLPTNDTVSFEYLKATVNPGIIFNITVHPDASGKYPELTYIKRICKNFPTDSNVQGHIIGMCYNAEWQFSSTPTCPASGCPPLPDDGIVFYEYYGYAGDRHTVGPVVTKDSSGNYPSPTHARRRCRALSQEADPGEFVAICYKSGTTGESHWEYYKNIGKCPDPRCKPLEANESVHYEYFTMTNETDKKKGPPAKVGKSGKYPEHTCVKKVCSKWPYTCSAGGPIFGECIGATWNFTALMECINARGCDENDLFELGFKEIMVREGEFSDSYRDAYVLFYATGSKVSAQCRGETARLECSNGEWHDLGAKTVHRAQRKDFAHHEEYFLILE* |
| **TGM-4** | MG429739 | MLLIVLIGLLEVAATDASGCMPFSDETASYKYLTERSRNDETPAQNDSSGAYPDHTHVKRFCKGLHGEEKTGRYVGICLGSEWVYYQGVQECQDRRCSPLPTNDTVTYEYLKATVNAGINFNITVHPDASGKYPELTYIKRICKNFPADSKVQGHIIGMCYNAEWRFSSTPTCPPSGCPPLPDDGIVFYEYYGYAGNRHTVGRAVSKDSSGNYPPQTHARRRCRALSQKADPGEFVGICYKSGTTGESHWDYYSHIRKCPDPRCKPLETNVSVHYEYFTMTNETGRKEGTPAEVDKGGKYPQHTCVRKFCDKSPYTCSVKGPIFGECLDGQWNFTALDECLNARGCNSDDLFDKLGFEGVMVREEEGSDSYKDDFVRFYATGSKVNAECKGKTVQLECSDGEWHDPGTKTVHRCTKEGIRAL* |
| **TGM-5** | MG429740 | MLLTVVIGLLEVAATDDSGCMPFSDEAATYKYVAKGPKNIEIPAQIDNSGMYPDYTHVKRFCKGLHGEDTTGWFVGIGPGSEWVYYQGVQECGGRGCLPLSTDDTVTFEYLKATVNPGIIFNITVHPDATGKYPELTYIKRICKNFPTDSKVQGHIIGMCYNAEWRFSSTPTSPSIGCPPLPDDGIVFHEYYGYAGDRHTVGPAVTKDSSGNYPSPTHARRRCRALSQKADPGEFVGICYRCGTTGESHWDYYSHIRKCPDPRGCNSDDLFDKLGFEGVMVREEEGSDSYKDDFARFYATGSKVNAECKGNTVRLECSNGEWHDPGTKTVHRCTKEGLRAP* |
| **TGM-6** | MG429741 | MLSLFIAIGLLEAAGSSCPPLPDDETVWYEYYGYVDGRHTVGDAAIKDSLENYPPNTHARRHCKALSKKADPGEFVAICYQRRGTSESQWQYYPRIASCPDPRCKPLEKNDSVSYEYFTKPTKGLKMGSITKPDKSGKYPEETFVRRYCNDLPRNSLAQGKTYAECLDSEWKLKNLPDCRFAAGCDEEYLLEKLMFVDISYWGKDAAKFSDDKTYRYYRPGSKVTAKCKGKSVKLTCVDGGYWVTVDGRKALCT* |
| **TGM-7** | MG429742 | MLLIVVIGLLEASAAGDNSCMPLSEETDTYQYFAQTSNKEETPARKDSSGMYPEYTHVKRFCKGLHGEDKTGEFIGLCHRSEWVYYMGVQECRDRRCSPLSESDTVSYEYRKATLNSSRISYDTSANPDDSGKYPELTYIRRICKNFPANSKVRGVIVGMCYNAEWRFSSAPVCPPYGCPPIQDNDKFRHEYYEYAGSRDVVGQAVTKDSSGNYPPQTHARRHCQARSVKVDQGELVAICHERSDTGESRWVYYHNIKQCPVPGCTITYKANSTVTYKYLQYTMSPDQTVVAKEATPQSGEYPDGTFAIVICKIPTRIGEKTGTIIAQCSNRQWKPEKYQIIPECPGRECAPLTDNDTVRYEYLNISRTGYYTLSTSTVKPRSGKYPERTSAKMFCKKATGDNKNLGEIAGRCSKGEWVKENEATVLQCPILGCLPLAENDTVEYKYFKSEQTSNDFANIAVLDWSGKFAVGSYARRICKKLDVNSRAQGDISKCGANGWVHRNTWPCPPQGSCNINEVYLDLKFTSTIVDTRLAVAVYKKPDTVSTFYGPGSKIQALCKGNPADLECFEGGWQGRRHAENERKFAKIRCTDSGVTYDE* |
| **TGM-8** | MG429743 | MLLIVVIGLLEASAAGDNSCMPLSEETDTYQYFAQTSNKEETPARKDSSGMYPEYTHVKRFCKGLHGEDKTGEFIGLCHRSEWVYYMGVKECRDRRCSPLSESDTVSYEYRKATLNSSRISYDTNANPDSSGKYPELTYIRRTCKNFSVDSKIRGLIVGMCYNAEWRFSSTPECPPYGCPPIQDNDNFRHEYYKYAGSREKLGPAATKDSSGNYPPQTHARRRCSAGSGKDGQGEFVAICLERDSTGESRWVYYLNIKQCPDPGCTTAYEDNSTVTYKYLQYTISPDQTVVAKEATPQGGEYPDGTFAIVICKIPTRIGEKTGTIFAQCSNRKWKPEKYQIVPECPGRECAPLTDNDTVRYEYLNISRTGYYTLSTSKVKPRSGKYPEGTSAKMFCKKATDDNKNLGEIAGRCSKGEWVKENEATVLQCPILGCLPLHENDTVEYKYFKSEHPNIAHENIAPLDWSGKFAVGSYAWRICKELDEKSEAQGDISKCGENGWEHSNTWPCPPRGSCDISDVFLDQKFTSTIVHTSLAVAVYQKPGTVSPYYGPGSKIQALCKGNPADLECFEGGWQGRRHAENERKFAKIRCTDSGVTYDE* |
| **TGM-9** | MG429744 | MLLLFVAIGLVEAVGSSCLPLPDNETVWYEYYEYVENRHTVGEAATKDTSGNYPPQTHARAHCKALSKKADPGVFVAICYQRRGSQWMYYRNITACPDPRCEPLKKSVSVSYEYYTKAAEGKGMGTLTNPDGSGKYPEQTLVRRYCNELPRNSLAQAETYAECLDSEWKLKNLPDCRFAAGCDEEYLLEKLMFSDISYWVNQPAKFSEDNTYRHYRPGSRVTAKCKGESVKLTCADGGHWVTADGRKALCE* |
| **TGM-10** | MG429745 | MWFSLIAVAVFNVAGASDGCLPLSEETATYEYYAYSGSRYVDGNPTEKDSSGRYPHGTHAKRFCKGSDEEAGLFVAICVKYRWVYYKDVKPCPDFRCQPLTPNETISNYQYLKETTNSGGESFEVVQPDADGKYPELTYIRRTCNEFPTDRKLQRDIAGLCYKAEWFLRTCPTPGNCYDDDIRTKLKYQGYSFDYETAEVTYSFGNDGAHYFIEGSQVTGICNGYQVPLWCQDGEWIGEVKNISCDMMNAQ* |

**Supplementary Table S2**

Nucleotide sequences of *Heligmosomoides polygyrus* Transforming Growth factor-β Mimic (TGM) family members, codon optimised for expression in human HEK293T cells. Inserts were synthesized by GeneArt with flanking sequences for cloning into the pSecTag2A plasmid vector, downstream of a 5' vector-encoded ATG start codon and Ig Kappa chain signal peptide prior to an *Asc*I restriction site (GG^CGCGCC). The 3' end of the synthetic insert contained an *Apa*I restriction site (GGGCC^C) for in-frame insertion reading through to Myc and 6-His tags, before a TAA stop codon. Sequences below represent the inserts between the two restriction sites.

| Gene Name | Sequence of Protein Optimised | Nucleotide Sequence |
| --- | --- | --- |
| **TGM-1** | 16-422  (1221 bp) | GATGATAGCGGCTGCATGCCTTTTAGCGACGAGGCCGCCACCTACAAATACGTGGCCAAGGGACCCAAGAACATCGAGATCCCCGCCCAGATCGACAACAGCGGCATGTACCCCGACTACACCCACGTGAAGCGGTTCTGCAAGGGCCTGCACGGCGAGGATACCACCGGATGGTTCGTGGGCATCTGCCTGGCCAGCCAGTGGTACTACTACGAGGGCGTGCAGGAATGCGACGACAGAAGATGCAGCCCCCTGCCCACCAACGACACCGTGTCCTTCGAGTACCTGAAGGCCACCGTGAACCCCGGCATCATCTTCAACATCACCGTGCACCCTGACGCCTCCGGCAAGTACCCTGAGCTGACCTACATCAAGAGGATCTGCAAGAACTTCCCCACCGACAGCAACGTGCAGGGCCACATCATCGGCATGTGCTACAACGCCGAGTGGCAGTTCAGCAGCACCCCTACCTGTCCTGCCAGCGGCTGTCCTCCCCTGCCTGATGATGGCATCGTGTTCTACGAGTACTACGGCTACGCCGGCGACCGGCACACAGTGGGACCTGTCGTGACCAAGGACAGCAGCGGCAACTACCCCAGCCCTACACATGCCAGACGCCGGTGTAGAGCCCTGAGCCAGGAAGCCGATCCTGGCGAGTTTGTGGCCATCTGCTACAAGAGCGGCACCACCGGCGAGAGCCACTGGGAGTACTACAAGAATATCGGCAAGTGCCCCGACCCCCGGTGCAAGCCTCTGGAAGCCAATGAGAGCGTGCACTACGAGTATTTCACCATGACCAACGAGACAGACAAGAAGAAGGGACCCCCTGCCAAAGTGGGCAAGAGCGGAAAGTACCCCGAGCACACCTGTGTGAAGAAAGTGTGCAGCAAGTGGCCCTACACCTGTAGCACCGGCGGACCCATCTTCGGCGAGTGTATCGGCGCCACCTGGAACTTCACCGCCCTGATGGAATGCATCAACGCCAGAGGCTGCAGCAGCGACGACCTGTTCGACAAGCTGGGCTTCGAGAAAGTGATCGTGCGGAAGGGCGAGGGCAGCGACAGCTACAAGGACGACTTCGCCCGGTTCTACGCCACCGGCAGCAAAGTGATTGCCGAGTGCGGCGGCAAGACCGTGCGGCTGGAATGTAGCAATGGCGAGTGGCACGAGCCCGGCACCAAGACAGTGCACAGATGCACCAAGGACGGCATCCGGACCCTG |
| **TGM-2** | 16-430  (1249 bp) | CCGATGATAGCGGCTGCATGCCTTTTAGCGACGAGGCCGCCACCTACAAATACGTGGCCAAGGGCCCCAAGAACATCGAGATCCCCGCCCAGATCGACAACAGCGGCATGTACCCCGACTACACCCACGTGAAGCGGTTCTGCAAGGGCCTGCACGGCGAGGATACCACCGGATGGTTCGTGGGCATCTGCCTGGCCAGCCAGTGGTACTACTACGAGGGCGTGCAGGAATGCGACGACAGAAGATGCAGCCCCCTGCCCACCAACGACACCGTGTCCTTCGAGTACCTGAAGGCCACCGTGAACCCCGGCATCATCTTCAACATCACCGTGCACCCTGACGCCTCCGGCAAGTACCCTGAGCTGACCTACATCAAGAGGATCTGCAAGAACTTCCCCACCGACAGCAACGTGCAGGGCCACATCATCGGCATGTGCTACAACGCCGAGTGGCAGTTCAGCAGCACCCCTACCTGTCCTGCCAGCGGCTGTCCTCCCCTGCCTGATGATGGCATCGTGTTCTACGAGTACTACGGCTACGCCGGCGACAGACACACAGTGGGCCCTGTCGTGACCAAGGACAGCAGCGGCAACTACCCCAGCCCTACACATGCCAGACGCCGGTGTAGAGCCCTGAGCCAGAAAGCCGATACCGGCGAGTTTGTGGCCATCTGCTACAAGAGCTGCACCACCGGCGAGAGCCACTGGCAGTACTACAAGTATATCAAGAACTGCCCCGACCCCCGGTGCAAGCCTCTGAAGGCCGATGAGTCCGTCAGATACGAGTATTTCACCATGGCCAACGAGACAGGCAAGAAAGAGGGCACACCCGCCCAGGTGGACAAGGGCGGCAAGTATAGCCAGCACACCTGTGTGCGGAAGTTCTGCGACAAGTCCCCCTACACCTGTAGCGTGAAGGGCCCTATCTTCGGCGAGTGCCTGGATGGCCAGTGGAACTTCACCGCCCTGGATGAGTGCCTGAACGCCAGAGGATGTGACGGCGGCGACCTGTTCAACAAGCTGGGCTTCGAGATCGTGATGGTGCGCGAGGGCGAGGGCAGCGACAGCTACAAGGATGACTACGTGCGGTTCTACACAACCGGCAGCAAAGTGAATGCCGAGTGCAAGGGCAAGACCGTGCGGCTGGAATGCAGCAATGGCGAGTGGCACGACTCCGAGACACGGACCGTGCACAGATGTACCAGCGAGGGCATCCGGCACTATGAGGGCTACAGCCTGATCCTGGAAGC |
| **TGM-3** | 17-429  (1246 bp) | CCGATGATAGCGGCTGCATGCCTTTTAGCGACGAGGCCGCCACCTACAAATACGTGGCCAAGGGCCCCAAGAACATCGAGATCCCCGCCCAGATCGACAACAGCGGCATGTACCCCGACTACACCCACGTGAAGCGGTTCTGCAAGGGCCTGCACGGCGAGGATACCACCGGATGGTTCGTGGGCATCTGCCTGGCCAGCCAGTGGTACTACTACGAGGGCGTGCAGGAATGCGACGACAGAAGATGCAGCCCCCTGCCCACCAACGACACCGTGTCCTTCGAGTACCTGAAGGCCACCGTGAACCCCGGCATCATCTTCAACATCACCGTGCACCCTGACGCCTCCGGCAAGTACCCTGAGCTGACCTACATCAAGAGGATCTGCAAGAACTTCCCCACCGACAGCAACGTGCAGGGCCACATCATCGGCATGTGCTACAACGCCGAGTGGCAGTTCAGCAGCACCCCTACCTGTCCTGCCAGCGGCTGTCCTCCCCTGCCTGATGATGGCATCGTGTTCTACGAGTACTACGGCTACGCCGGCGACAGACACACAGTGGGCCCTGTCGTGACCAAGGACAGCAGCGGCAACTACCCCAGCCCTACACATGCCAGACGCCGGTGTAGAGCCCTGAGCCAGGAAGCCGATCCTGGCGAGTTTGTGGCCATCTGCTACAAGAGCGGCACCACCGGCGAGAGCCACTGGGAGTACTACAAGAATATCGGCAAGTGCCCCGACCCCCGGTGCAAGCCTCTGGAAGCCAATGAGAGCGTGCACTACGAGTATTTCACCATGACCAACGAGACAGACAAGAAGAAGGGCCCTCCCGCCAAAGTGGGCAAGAGCGGAAAGTACCCCGAGCACACCTGTGTGAAGAAAGTGTGCAGCAAGTGGCCCTACACCTGTTCTGCCGGCGGACCCATCTTCGGCGAGTGTATCGGCGCCACCTGGAACTTCACCGCCCTGATGGAATGCATCAACGCCAGAGGCTGCGACGAGAACGACCTGTTCGAGCTGGGCTTCAAAGAAATCATGGTGCGCGAGGGCGAGTTCAGCGACAGCTACAGAGATGCCTACGTGCTGTTCTACGCCACCGGCAGCAAAGTGTCCGCCCAGTGCAGAGGCGAGACAGCCAGACTGGAATGCAGCAATGGCGAGTGGCACGACCTGGGCGCCAAGACAGTGCACAGAGCCCAGAGAAAGGACTTCGCCCACCACGAGGAATACTTTCTGATCCTGGAAGC |
| **TGM-4** | 17-422  (1222 bp) | GCGCCTCTGGCTGCATGCCTTTCAGCGACGAGACAGCCAGCTACAAGTACCTGACCGAGCGGAGCCGGAACGACGAAACCCCTGCCCAGAATGATAGCAGCGGCGCCTACCCCGATCACACCCACGTGAAGAGATTCTGCAAGGGCCTGCACGGCGAGGAAAAGACCGGCAGATACGTGGGCATCTGCCTGGGCAGCGAGTGGGTGTACTATCAGGGCGTGCAGGAATGCCAGGACAGACGGTGTAGCCCCCTGCCTACCAACGACACCGTGACCTACGAGTATCTGAAGGCCACCGTGAACGCCGGCATCAACTTCAACATCACCGTGCACCCCGACGCCAGCGGCAAGTATCCTGAGCTGACCTACATCAAGCGGATCTGCAAGAACTTCCCCGCCGACAGCAAGGTGCAGGGCCACATCATCGGCATGTGCTACAACGCCGAGTGGCGGTTCAGCAGCACCCCTACCTGTCCTCCAAGCGGCTGTCCCCCTCTGCCCGATGATGGCATCGTGTTCTACGAGTACTACGGCTACGCCGGCAACCGGCACACAGTGGGCAGAGCCGTGTCCAAGGACAGCTCCGGCAACTACCCTCCTCAGACCCATGCCAGACGCCGGTGTAGAGCCCTGAGCCAGAAAGCCGATCCCGGCGAGTTCGTGGGAATCTGCTACAAGAGCGGCACCACCGGCGAGAGCCACTGGGACTACTACAGCCACATCCGGAAGTGCCCCGACCCCAGATGCAAGCCCCTGGAAACAAACGTGTCCGTGCACTATGAGTACTTCACCATGACCAACGAGACAGGCCGGAAAGAGGGCACCCCTGCCGAAGTGGATAAGGGCGGAAAGTACCCCCAGCACACCTGTGTGCGGAAGTTCTGCGACAAGTCCCCCTACACCTGTAGCGTGAAGGGCCCCATCTTCGGCGAGTGCCTGGACGGCCAGTGGAACTTCACAGCCCTGGATGAGTGCCTGAATGCCAGAGGCTGCAACAGCGACGACCTGTTCGACAAGCTGGGCTTCGAGGGCGTGATGGTGCGCGAGGAAGAGGGCAGCGACTCCTACAAGGACGACTTCGTGCGGTTCTACGCCACCGGCAGCAAAGTGAATGCCGAGTGCAAGGGCAAGACCGTGCAGCTGGAATGCAGCGACGGCGAGTGGCACGATCCTGGCACCAAGACAGTGCACAGATGCACCAAAGAGGGAATCCGGGCCCTGGC |
| **TGM-5** | 16-341  (982 bp) | CCGATGATAGCGGCTGCATGCCTTTTAGCGACGAGGCCGCCACCTACAAATACGTGGCCAAGGGCCCCAAGAACATCGAGATCCCCGCCCAGATCGACAACAGCGGCATGTACCCCGACTACACCCACGTGAAGCGGTTCTGCAAGGGCCTGCACGGCGAGGATACCACCGGATGGTTCGTGGGCATCGGCCCTGGAAGCGAGTGGGTGTACTATCAGGGCGTGCAGGAATGCGGCGGCAGAGGCTGTCTGCCTCTGAGCACCGATGACACCGTGACCTTCGAGTACCTGAAGGCCACCGTGAACCCCGGCATCATCTTCAACATCACCGTGCACCCTGACGCCACCGGCAAGTACCCTGAGCTGACCTACATCAAGAGGATCTGCAAGAACTTCCCCACCGACAGCAAGGTGCAGGGCCACATCATCGGCATGTGCTACAACGCCGAGTGGCGGTTCAGCAGCACCCCTACCAGCCCTAGCATCGGCTGTCCCCCCCTGCCTGATGACGGCATCGTGTTCCACGAGTACTACGGCTACGCCGGCGACAGACACACAGTGGGACCTGCCGTGACCAAGGACAGCAGCGGCAACTACCCTAGCCCCACACATGCCAGACGCCGGTGTAGAGCCCTGAGCCAGAAAGCCGATCCCGGCGAGTTTGTGGGCATCTGCTACAGATGTGGCACCACCGGCGAGAGCCACTGGGACTACTACAGCCACATCCGGAAGTGCCCCGACCCCAGAGGCTGCAACAGCGACGACCTGTTCGACAAGCTGGGCTTCGAGGGCGTGATGGTGCGCGAGGAAGAGGGCAGCGACAGCTACAAGGACGACTTCGCCCGGTTCTACGCCACAGGCAGCAAAGTGAATGCCGAGTGCAAGGGCAACACCGTGCGGCTGGAATGCAGCAATGGCGAGTGGCACGACCCCGGCACCAAGACCGTGCACAGATGCACCAAAGAGGGCCTGAGAGCCCCTGC |
| **TGM-6** | 17-254  (718 bp) | CCTCTTGTCCTCCCCTGCCCGATGATGAGACAGTGTGGTACGAGTACTACGGCTACGTGGACGGCAGACACACAGTGGGCGACGCCGCCATCAAGGACAGCCTGGAAAACTACCCCCCCAACACCCACGCCAGACGGCACTGTAAAGCCCTGAGCAAGAAGGCCGACCCCGGCGAGTTTGTGGCCATCTGCTACCAGAGAAGAGGCACCAGCGAGAGCCAGTGGCAGTACTACCCCAGAATCGCCAGCTGCCCCGACCCCAGATGCAAGCCCCTGGAAAAGAACGACAGCGTGTCCTACGAGTATTTCACCAAGCCCACCAAGGGCCTGAAGATGGGCAGCATCACAAAGCCCGACAAGAGCGGCAAGTACCCCGAAGAGACATTCGTGCGGCGGTACTGCAACGACCTGCCCAGAAATTCTCTGGCCCAGGGCAAGACCTACGCCGAGTGCCTGGATAGCGAGTGGAAGCTGAAGAACCTGCCCGACTGCAGATTCGCCGCTGGCTGCGACGAGGAATACCTGCTGGAAAAGCTGATGTTCGTGGACATCAGCTACTGGGGCAAGGATGCCGCCAAGTTCAGCGACGATAAGACCTACCGGTACTACAGACCCGGCAGCAAAGTGACCGCCAAGTGCAAGGGCAAGAGCGTGAAGCTGACCTGTGTGGACGGCGGCTACTGGGTCACCGTGGATGGAAGAAAGGCCCTGTGCACAGC |
| **TGM-7** | 17-599  (1753 bp) | CCGACAATAGCTGCATGCCCCTGAGCGAGGAAACCGACACCTACCAGTACTTCGCCCAGACCAGCAACAAAGAGGAAACCCCCGCCAGAAAGGACAGCAGCGGCATGTACCCCGAGTACACCCACGTGAAGCGGTTCTGCAAGGGCCTGCACGGCGAGGATAAGACCGGCGAGTTCATCGGCCTGTGCCACAGATCCGAATGGGTGTACTACATGGGCGTGCAGGAATGCCGGGACAGAAGATGCAGCCCCCTGTCCGAGAGCGACACCGTGTCCTACGAGTACCGGAAGGCCACCCTGAACAGCAGCCGGATCAGCTACGACACCAGCGCCAACCCCGACGACTCCGGCAAGTATCCCGAGCTGACCTACATCCGGCGGATCTGCAAGAACTTCCCCGCCAACAGCAAAGTGCGGGGCGTGATCGTGGGCATGTGCTACAATGCCGAGTGGCGGTTCAGCAGCGCCCCTGTGTGTCCTCCTTACGGCTGCCCCCCCATCCAGGACAACGACAAGTTCCGGCACGAGTACTACGAGTATGCCGGCAGCAGGGACGTCGTGGGACAGGCCGTGACAAAGGACTCCAGCGGCAACTACCCCCCCCAGACACACGCCAGAAGGCACTGTCAGGCCCGCAGCGTGAAAGTGGATCAGGGCGAGCTGGTGGCCATCTGCCACGAGAGAAGCGACACAGGCGAGAGCCGCTGGGTGTACTATCACAACATCAAGCAGTGCCCCGTGCCCGGCTGCACCATCACCTACAAGGCCAACTCCACCGTGACATACAAGTACCTGCAGTACACCATGAGCCCCGACCAGACCGTGGTGGCCAAAGAGGCCACACCTCAGAGCGGCGAGTACCCTGATGGCACCTTCGCCATCGTGATCTGCAAAATCCCCACCCGGATCGGCGAGAAAACCGGCACCATCATTGCCCAGTGCAGCAACCGGCAGTGGAAGCCCGAGAAGTACCAGATCATCCCCGAGTGCCCCGGCAGAGAGTGTGCCCCCCTGACCGACAACGATACCGTCAGATATGAGTACCTGAACATCAGCAGGACCGGGTACTACACCCTGAGCACCAGCACCGTGAAGCCCAGAAGCGGAAAGTACCCAGAGCGGACCTCCGCCAAGATGTTCTGCAAAAAGGCCACCGGCGACAACAAGAACCTGGGCGAGATCGCCGGCAGATGCAGCAAAGGCGAGTGGGTCAAAGAAAACGAGGCCACCGTGCTGCAGTGCCCTATCCTGGGATGTCTGCCCCTGGCCGAGAATGACACAGTGGAGTACAAGTACTTCAAGAGCGAGCAGACCTCCAACGACTTCGCCAATATCGCCGTGCTGGATTGGAGCGGCAAGTTCGCCGTGGGCAGCTACGCTCGGAGAATCTGTAAAAAGCTGGACGTGAACTCCAGAGCCCAGGGCGACATCAGCAAGTGCGGCGCCAATGGCTGGGTGCACAGAAACACCTGGCCATGCCCACCACAAGGCAGCTGCAACATCAACGAGGTGTACCTGGACCTGAAGTTCACCTCCACCATCGTGGACACCAGACTGGCCGTGGCCGTGTACAAGAAACCCGATACCGTGTCCACCTTCTACGGCCCTGGCAGCAAGATCCAGGCCCTGTGCAAGGGCAACCCCGCCGACCTGGAATGTTTTGAGGGCGGCTGGCAGGGAAGAAGGCACGCCGAGAACGAGAGAAAGTTCGCCAAGATCAGATGCACCGACAGCGGCGTGACCTACGACGAAGC |

**Supplementary Table S3**

Primers used for PCR amplification of domain-truncated *Heligmosomoides polygyrus* Transforming Growth factor-β Mimic (TGM)-1 proteins. For each primer, the gcgcgc cap is shown in lower case bold; the restriction sites (GG^CGCGCC for *Asc*I and GGGCC^C for *Apa*I) in upper case bold, and the sequence corresponding to the domain to be amplified in plain lower case. The third column gives the native *H. polygyrus* nucleotide (nt) sequence for the same segment. The right-hand columns denote the nucleotide positions in the full-length TGM-1 open reading frame to which these sequences correspond, and finally the amino acid positions in the full-length TGM-1 protein. Amino acid positions are given 5' to 3' for both 5'-3' and 3'-5' primers.

| **Primer Name** | **5’ – 3’** | **Native nt Sequence** | **Nucleotide Positions** | **Amino Acid Positions** |
| --- | --- | --- | --- | --- |
| coTGM_domain1F | **gcgcgcGGCGCGCC**gatgatagcggctgcatg | gatgacagcggctgcatg | 46-63 | 16-21  DDSGCM |
| coTGM_domain2F | **gcgcgcGGCGCGCC**agatgcagccccctgccc | aggtgctcgccgttgccga | 286-303 | 96-101  RCSPLP |
| coTGM_domain3F | **gcgcgcGGCGCGCC**ggctgtcctcccctgcctg | ggttgcccaccgctgccag | 529-547 | 177-182  GCPPLP |
| coTGM_domain4F | **gcgcgcGGCGCGCC**cggtgcaagcctctggaag | agatgcaagccactggaag | 787-805 | 263-268  RCKPLE |
| coTGM_domain5F | **gcgcgcGGCGCGCC**agaggctgcagcagcgacg | agaggttgtagctcagacg | 1030-1048 | 344-349  RGCSSD |
|  | **3’ — 5’** | **3’ — 5’** | **3’ — 5’** | **3’ — 5’** |
| coTGM_domain1R | **gcgcgcGGGCCC**tctgtcgtcgcattcctgcac | acgatcatcgcattcttgtac | 285-265 | 95-89  VQECDDR |
| coTGM_domain2R | **gcgcgcGGGCCC**gctggcaggacaggtag | actggctgggcatgttg | 528-512 | 176-172  TCPAS |
| coTGM_domain3R | **gcgcgcGGGCCC**ggggtcggggcacttgcc | aggatctgggcactttcc | 786-769 | 262-257  GKCPDP |
| coTGM_domain4R | **gcgcgcGGGCCC**ggcgttgatgcattccatcag | tgcgttaatgcattccataag | 1029-1009 | 343-337  LMECINA |
| coTGM_domain5R | **gcgcgcGGGCCC**cagggtccggatgcc | tagtgtgcgaattcc | 1266 - 1252 | 422-418  GIRTL |
