# Supplementary figures and images for: TGF-β mimic proteins form an extended gene family in the murine parasite Heligmosomoides polygyrus
Source: Int J Parasitol. 2018 Apr;48(5):379–85. doi: 10.1016/j.ijpara.2017.12.004 (PMC5904571; doi:10.1016/j.ijpara.2017.12.004)

**Supplementary Fig. S1.**

**
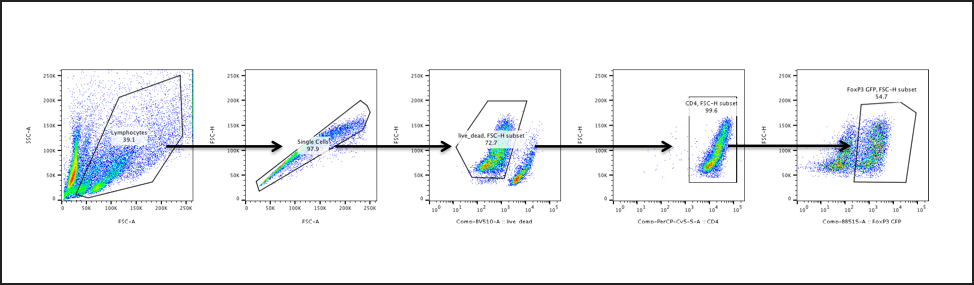
**

Supplement: Supplementary Fig. S1 [file mmc2.docx]
